# Biodistribution and Tolerability of AAV-PHP.B-CBh-*SMN1* in Wistar Han Rats and Cynomolgus Macaques Reveal Different Toxicologic Profiles

**DOI:** 10.1089/hum.2021.116

**Published:** 2022-02-14

**Authors:** Xavier Palazzi, Ingrid D. Pardo, Madhu P. Sirivelu, Leah Newman, Steven W. Kumpf, Jessie Qian, Tania Franks, Sarah Lopes, June Liu, Laura Monarski, Sandra Casinghino, Casey Ritenour, Hayley Ritenour, Christopher Dubois, Jennifer Olson, John Graves, Kristin E. Alexander, Timothy Coskran, Thomas A. Lanz, Joseph Brady, Douglas McCarty, Somanathan Suryanarayan, Laurence O. Whiteley

**Affiliations:** ^1^ Drug Safety Research and Development, Pfizer Worldwide Research Development and Medicine, Groton, Connecticut, USA.; ^2^ Drug Safety Research and Development, Pfizer Worldwide Research Development and Medicine, Cambridge, Massachusetts, USA.; ^3^ Rare Diseases Research Unit, Pfizer Worldwide Research Development and Medicine, Cambridge, Massachusetts, USA.

**Keywords:** adeno-associated virus, biodistribution, liver, dorsal root ganglia, toxicity, gene therapy, transduction

## Abstract

Recombinant adeno-associated viruses (AAVs) have emerged as promising vectors for human gene therapy, but some variants have induced severe toxicity in Rhesus monkeys and piglets following high-dose intravenous (IV) administration. To characterize biodistribution, transduction, and toxicity among common preclinical species, an AAV9 neurotropic variant expressing the survival motor neuron 1 (*SMN1*) transgene (AAV-PHP.B-CBh-*SMN1*) was administered by IV bolus injection to Wistar Han rats and cynomolgus monkeys at doses of 2 × 10^13^, 5 × 10^13^, or 1 × 10^14^ vg/kg. A dose-dependent degeneration/necrosis of neurons without clinical manifestations occurred in dorsal root ganglia (DRGs) and sympathetic thoracic ganglia in rats, while liver injury was not observed in rats. In monkeys, one male at 5 × 10^13^ vg/kg was found dead on day 4. Clinical pathology data on days 3 and/or 4 at all doses suggested liver dysfunction and coagulation disorders, which led to study termination. Histologic evaluation of the liver in monkeys showed hepatocyte degeneration and necrosis without inflammatory cell infiltrates or intravascular thrombi, suggesting that hepatocyte injury is a direct effect of the vector following hepatocyte transduction. *In situ* hybridization demonstrated a dose-dependent expression of *SMN1* transgene mRNA in the cytoplasm and DNA in the nucleus of periportal to panlobular hepatocytes, while quantitative polymerase chain reaction confirmed the dose-dependent presence of *SMN1* transgene mRNA and DNA in monkeys. Monkeys produced a much greater amount of transgene mRNA compared with rats. In DRGs, neuronal degeneration/necrosis and accompanying findings were observed in monkeys as early as 4 days after test article administration. The present results show sensory neuron toxicity following IV delivery of AAV vectors at high doses with an early onset in Macaca fascicularis and after 1 month in rats, and suggest adding the autonomic system in the watch list for preclinical and clinical studies. Our data also suggest that the rat may be useful for evaluating the potential DRG toxicity of AAV vectors, while acute hepatic toxicity associated with coagulation disorders appears to be highly species-dependent.

## INTRODUCTION

Engineered adeno-associated viruses (AAVs) have become very popular vectors for gene therapy owing to the apparent lack of pathogenicity of their natural counterparts.^1,2^ Use of tissue-specific promoters to restrict transgene expression and the natural tropism of viral capsids to selective tissues has been exploited to restore factor VIII or IX expression in hepatocytes, neurons, or myofibers for specific disease entities such a spinal muscular atrophy or Duchenne's muscular dystrophy.^3,4^ However, safety concerns have been raised for some AAV vectors administered in animal species and in humans.^5–7^ In particular, acute liver failure with marked hepatic enzyme elevations and systemic inflammatory response syndromes in animals and humans, sensory neuron degeneration/necrosis in animals, and concerns regarding insertional mutagenesis in animals have been raised.^8^

The objective of our study was to determine if the AAV-induced hepatic and neuronal toxicity reported by others^5^ in Rhesus monkeys could be reproduced in cynomolgus monkeys and Wistar Han rats, which are commonly used in nonclinical safety assessment of drugs. We used a similar AAV9 variant vector (AAV-PHP.B) that expressed the human survival motor neuron 1 (*SMN1*) transgene driven by the ubiquitous CBh (hybrid form of chicken beta actin) promoter and similar doses across the two species.

## MATERIALS AND METHODS

### Animal procedures

These studies were conducted in accordance with the current guidelines for animal welfare. The procedures used in these studies have been reviewed and approved by Pfizer Institutional Animal Care and Use Committee.

Sixteen cynomolgus macaques of Mauritius origin, aged 2 years and 4 months to 4 years and weighing 3–4.9 kg, and seronegative (titer ≤1:5) for neutralizing antibodies (NAb) to the AAV-PHP.b capsid, were acclimated to the laboratory environment and outfitted for cardiovascular telemetry measurements (Supplementary Table S1). A vehicle consisting of 0.002% Pluronic F68 in 10 mM phosphate, 350 mM NaCl, 2.7 mM KCl, and 5% sorbitol in sterile water for injection, pH 7.4, was used to dilute the stock formulation to prepare the dosing formulations and to dose the control animals. Intravenous (IV) injection was performed in the saphenous vein on day 1 (Supplementary Table S2).

Wistar Han (Crl:WI[Han]) male rats were aged 7–9 weeks at study start. A single dose was administered by tail vein injection on day 1. The same vehicle was used as for the monkey study described above. All rats were fasted overnight before necropsy (Supplementary Table S3).

Both cynomolgus monkeys and rats were administered a bolus (∼1-min duration) IV injection of the AAV vector at 2 × 10^13^, 5 × 10^13^, or 1 × 10^14^ vg/kg from the same manufacture lot, at 2 and 5 mL/kg, respectively, and aligned with published best practices for dose-volume injections.^9^

Although toxicity data have been published with a similar construct at 2 × 10^14^ vg/kg in the rhesus monkey,^5^ establishing a dose–response capped at 10^14^ vg/kg in the cynomolgus monkey was needed to extrapolate these data in a species commonly used for regulatory toxicology, considering the variability of response across species^10^ and, for a given species, based on geographical origin.^11^

### Clinical pathology and biomarkers

In both studies, a core battery of hematology, coagulation, clinical chemistry, and additional biomarkers was assessed (Supplementary Tables S4 and S5 for monkeys and rats, respectively).

### Histopathology

Representative tissue samples were processed to slide and stained with hematoxylin and eosin. Slides were evaluated and peer reviewed by board-certified veterinary pathologists. Microscopic findings were graded on a scale of 1–5 as minimal, mild, moderate, marked, or severe (Supplementary Tables S6 and S7 for monkeys and rats, respectively). Formalin-fixed liver samples were sliced to 2 mm thickness and fixed in Karnovski's fixative at 4°C for transmission electron microscopy (TEM).

Liver in monkey and rat and dorsal root ganglia (DRGs) in monkey were collected for molecular endpoints.

### Neutralizing antibodies

A cell-based transduction inhibition assay was used to assess NAb titers. Briefly, AAV-PHP.b vectors expressing luciferase were admixed with heat-inactivated diluted serum for an hour at 37°C. Serum-complexed vector was added to HEK293 cells preinfected with wild-type human adenovirus 5 for 2 h to increase AAV transgene expression. Twenty-four hours later, cells were lysed, and luciferase expression quantified. Titers were reported as the lowest reciprocal serum dilution that resulted in >50% transduction inhibition.

### Binding antibodies

A plate-based enzyme-linked immunosorbent assay (ELISA) was used to evaluate binding antibodies (BAb). Briefly, vectors immobilized on ELISA plates overnight in phosphate-buffered saline were used to capture anti-AAV antibodies in serum. The following day, plates were washed and then blocked for an hour before adding diluted serum (1/100). Plates were incubated for an hour before washing and adding detection antibodies. BAb-positive sera were identified based on optical density values that were 10-fold higher compared to control wells.

### Vectors

AAV PHPb vectors contained the SMN transgene expressed from a CBh promoter, rabbit beta-globin (RBG) polyadenylation signal for termination, and AAV2 inverted terminal repeats. PHPb.CBh.SMN.RBG vectors were purified from HEK293 cells grown in suspension after triple transfection with the PHP.b packaging plasmid and adenovirus helper plasmid. Triple-transfected cells were lysed, crude lysates cleared, and vectors purified from affinity columns before quantifying vector genome titers.

### Immunohistochemistry

Standard methods were used for immunohistochemistry (IHC) on a Leica Bond Rx. Specific antibody and pretreatment conditions are listed in Supplementary Table S9.

### *In situ* hybridization

*In situ* hybridization (ISH) was performed using either the RNAscope 2.5 LS Assay—Brown reagent kit or the RNAscope 2.5 LSx Assay—Brown reagent kit according to the manufacturer's guidelines on a Leica Bond Rx. The human *SMN1* probe (Cat. No. 590908) was designed by Advanced Cell Diagnostics (Newark, CA) as a 16ZZ probe targeting base pairs 1982-2926 of the Pfizer *SMN1* sequence.

### DNase/RNase digestion for ISH

DNase I and RNase A digestions were completed offline after baking, deparaffinization, and epitope retrieval, two pretreatment steps of the ISH protocol on the Leica Bond Rx. DNase I was prepared using 10 mM Tris-HCl pH 7.5, 2.5 mM MgCl_2_, 0.5 mM CaCl_2_, and 80 μg/mL of deoxyribonuclease I, bovine recombinant, expressed in Pichia pastoris buffered aqueous glycerol solution (Cat. No. D5319; Sigma-Aldrich, St. Louis, MO). DNase I was applied to the tissue sections for 30 min at 37°C, and then rinsed in deionized water (3 × 2 min). RNase A was prepared using 10 mM Tris-HCl, pH 7.5, and 20 μg/mL of RNase A solution from bovine pancreas (Cat. No. R4642; Sigma-Aldrich) and applied to tissue sections for 10 min at room temperature followed by 20 min at 60°C, and then washed thoroughly in deionized water (3 × 2 min).

### Vector copy number and transgene quantification

DNA was isolated from all tissues using DNeasy Blood and Tissue kits following homogenization in Buffer ATL using a stainless-steel bead on a Tissue Lyser (Qiagen) for 2 min at 30 Hz. Tube sets were then rotated and this step was repeated for another 2 min. Homogenates were then incubated overnight at 56°C with proteinase K and applied the next day to QIAshredder columns (Qiagen) and centrifuged for 2 min at 20,000 *g*. Two hundred microliters of the lysate was loaded onto a QIAcube and downstream DNA isolation was performed using a Qiagen DNeasy blood and tissue protocol on a QIAcube according to manufacturer's instructions. Cynomolgus DNA concentrations were quantified by the Qubit Broad Range DNA assay (Life Technologies), and rat DNA concentrations were quantified by Big Lunatic (Unchained Labs).

To isolate RNA, tissues were homogenized in QIAzol lysis reagent (Qiagen) with a stainless-steel bead on a Tissue Lyser (Qiagen) for 2 min at 30 Hz. Tube sets were then rotated, and this step was repeated for another 2 min. Chloroform was added (200 mL/1,000 mL QIAzol), samples were shaken for 15 s, and then centrifuged at 12,000 *g* for 15 min at 4°C. The upper, aqueous phase was then transferred to a QIAcube and RNA isolation was performed using the Qiagen Mini protocol with DNase treatment on the QIAcube according to manufacturer's instructions.

RNA quality was assessed using a Tapestation 4200 instrument (Agilent). Cyno RNA concentration was measured by Nanodrop 1000 (Thermo Scientific), and rat RNA concentration was measured by Big Lunatic (Unchained Labs). RNA was reverse transcribed to cDNA using the High-Capacity RNA-to-cDNA kit (Life Technologies).

A custom TaqMan assay for the *SMN1* construct was designed using the intronic context sequence GGTGGCGGCAGGTGGGGGTGCCGGG for DNA, and GCCCTATTTGTGAGGTGGCCAACAA for transgene. Standard curves were created with the *SMN1* plasmid, ranging from 5 × 10^9^ copies to 5 copies in a log-dilution series. One hundred nanograms DNA or cDNA per sample was tested in triplicate in 96-well PCR plates with TaqMan Universal Master Mix II for a 20 mL reaction volume run on a ViiA7 Real-Time PCR System (Life Technologies). Viral genome copy number was interpolated for each DNA sample, or transgene copy number for each cDNA sample, using the plasmid standard curve.

### RNAseq evaluation of transgene expression in the liver

From RNA extracted from both the rat and cynomolgus monkey livers, RNAseq cDNA libraries were prepared from 1 μg total RNA using the Illumina TruSeq stranded mRNA kit (Cat. No. RS-122-2101) according to the manufacturer's instructions. Resulting libraries were sequenced on a NextSeq500 (Illumina) using a paired-end run (2 × 150 bases). A minimum of 10 million reads were generated from each library. Clean raw sequence reads in FASTQ were aligned to the *SMN1* transgene sequence CLC Workbench (Qiagen).

### Methods used for characterization of the administered vector

#### Host cell DNA quantitative polymerase chain reaction assay

The host cell DNA was measured using a sensitive, quantitative polymerase chain reaction (qPCR)-based method. The primers targeted a portion of the conserved human repeat sequence. The concentration of residual DNA in the samples was calculated from a standard curve prepared with human genomic DNA.

#### Residual plasmid DNA qPCR assay

Residual plasmid DNA was measured using a sensitive, qPCR-based method. The primers targeted a portion of an antibiotic resistance gene, ampicillin, contained in all the plasmids. The concentration of residual plasmid DNA in the samples was interpolated from a standard curve prepared with a plasmid standard.

#### Size exclusion chromatography

The test samples were injected onto a TOSOH TSKgel column and separated by isocratic elution. The viral particles (vp/mL) were determined using relative peak area quantitation against a calibration curve prepared with an AAV standard of a predetermined concentration. The chromatographic peak corresponding to the AAV was integrated at both 260 nm and 280 nm, and the peak area ratio (size exclusion chromatography UV260/280 ratio) was calculated for each sample.

#### Analytical ultracentrifugation sedimentation velocity analysis

Using a Beckman Coulter Optima Analytical Ultracentrifuge, a sedimentation velocity assessment was performed with interference data of 100 scans collected and analyzed using Sedfit to generate a c(s) distribution plot of empty and full capsid peaks. The c(s) peaks corresponding to the AAV particles between 55 and 105 S20w were integrated to determine relative abundance of empty and full capsids (Supplementary Table S8).

The vector was single stranded. The *SMN1* transgene was the native *SMN1* sequence and not codon optimized.

## RESULTS

### Cynomolgus monkey study

Test article-related mortality occurred on day 4. Male 6, administered 5 × 10^13^ vg/kg of AAV-PHP.b-CBh-*SMN1*, was found dead without prodromal clinical signs. A decision to euthanize all animals in this study was made based on clinical pathology findings suggestive of coagulopathy, including platelet decreases (down to a 0.06-fold baseline value, corresponding to 24,000 platelets/μL) and massive increases in ALT (up to a 40.7-fold baseline value, corresponding to 2,608 U/L).

Animals administered 5 × 10^13^ or 1 × 10^14^ vg/kg were euthanized on day 4, animals administered 2 × 10^13^ vg/kg on day 5, and controls on day 7. None displayed clinical signs.

**Figure 1.**
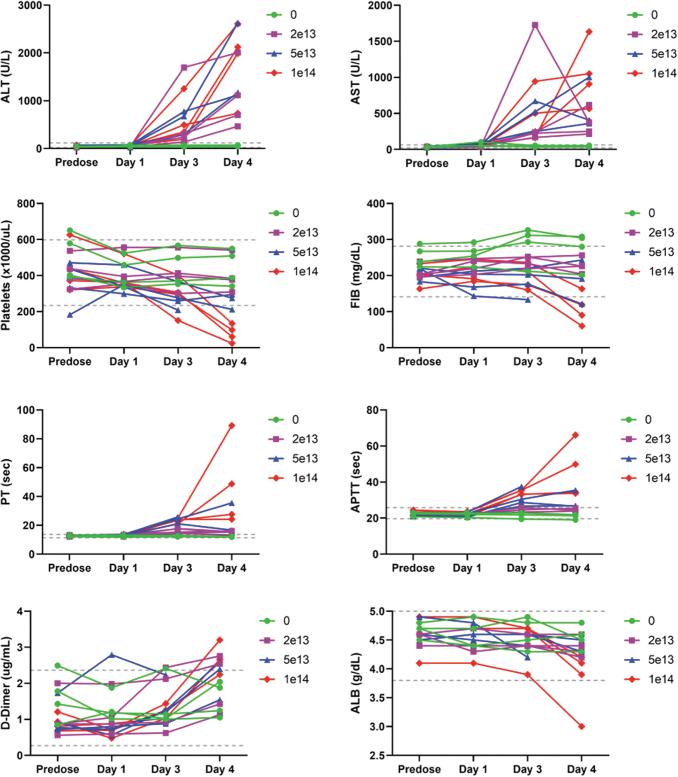
Composite figure showing individual treatment-related clinical pathology findings in ALT, AST, platelets, fibrinogen, PT, APTT, D-dimers, and albumin in cynomolgus monkeys. Individual data are given at *baseline* (predose) and on days 1, 3, and 4. Treatment with a single IV bolus injection of AAV-PHP.B-CBh-*SMN1* led to decreased platelets, fibrinogen, and albumin, and increased ALT, AST, PT, APTT, D-dimers, ALT, and AST. Reference intervals validated in the test facility are provided (*dashed line*). AAV, adeno-associated virus; IV, intravenous; SMN1, survival motor neuron 1.

Remarkable clinical pathology findings included decreased platelets, fibrinogen, and albumin, and increased PT, APTT, D-dimers, ALT, and AST (Fig. 1 and Supplementary Table S10).

**Figure 2.**
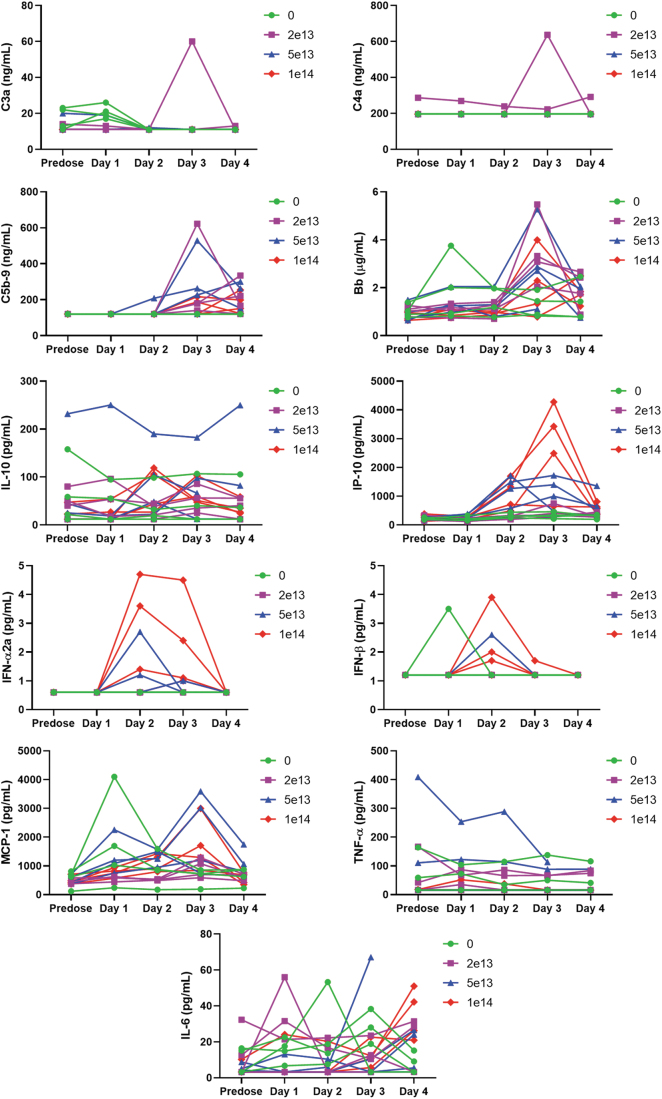
Composite figure showing individual treatment-related biomarker (complement C3a, C4a, C5b-9, and Bb) and cytokine (IL-10, IP-10, IFN-α, IFN-β, MCP-1, TNF-α, and IL-6) variations in cynomolgus monkeys receiving a single IV bolus injection of AAV-PHP.B-CBh-*SMN1*. Individual data are given at *baseline* (predose) and on days 1, 2, 3, and 4. The treatment induced no variation of C3a or C4a. C5b9, Bb, and MCP-1 spiked in most monkeys in a nondose-related manner. At the highest dose, IP-10 increased at 24 h postadministration and peaked 2 days after IV injection in a dose-related manner. Individual increases of IFN-α and IFN-β were observed in a similar time frame only at ≥5 × 10^13^ vg/kg. No dose-related variation of IL-10, IL-6, and TNF-α could be observed.

There was no variation of C3a or C4a, but C5b9, Bb, and MCP-1 spiked in most monkeys in a nondose-related manner before euthanasia. At ≥5 × 10^13^ vg/kg, IP-10 increased at 24 h postdose and peaked 2 days after IV administration in a dose-related manner, while individual increases of IFN-α and IFN-β were observed in a similar time frame only at ≥5 × 10^13^ vg/kg. No dose-related variation of IL-10, IL-6, and TNF-α could be observed (Fig. 2).

At necropsy, an abnormal color of the liver (both genders at ≥5 × 10^13^ vg/kg) correlated with degeneration/necrosis of hepatocytes and effusions in the abdominal and/or thoracic cavity.

**Figure 3.**
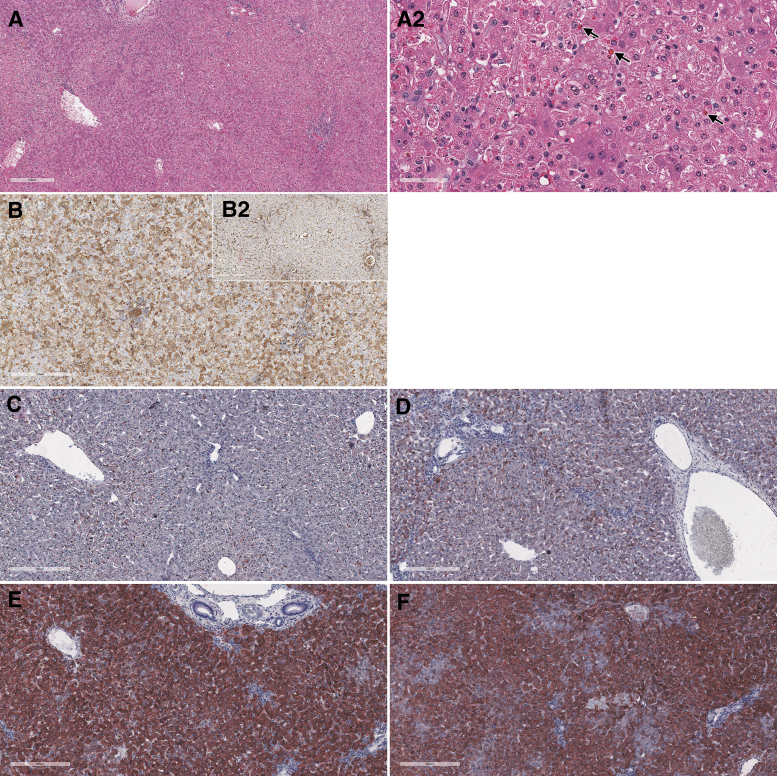
Liver from cynomolgus monkeys: composite figure depicting the morphologic changes observed microscopically in the liver of test article-treated versus control animals. Female 16 liver, 4 days after a single IV bolus injection of AAV-PHP.B-CBh-*SMN1* at 1 × 10^14^ vg/kg. The liver shows moderate hepatocellular degeneration and necrosis characterized by diffuse swelling and multifocal to coalescing necrosis of hepatocytes **(A)**. No specific lobular pattern can be identified. Staining: hematoxylin and eosin. Same animal at a higher magnification showing hepatocytic vacuolation, cellular disintegration, and the presence of intracytoplasmic eosinophilic inclusions of various sizes (*arrows*) **(A2)**. Albumin IHC in male 1 (control) **(***insert* **B2)** and male 7 **(B)** at 1 × 10^14^ vg/kg showing an increase in cytoplasmic staining of hepatocytes with a mosaic pattern. Oil red O staining of neutral lipids performed on frozen sections **(C–F)** shows a dose-related increase in lipid droplets in males 1, 3, 5, and 7 at 0, 2 × 10^13^, 5 × 10^13^, and 1 × 10^14^ vg/kg, respectively, suggesting hepatocellular malfunction subsequent to several possible mechanisms of toxicity described in the discussion section of this article. IHC, immunohistochemistry.

Remarkable microscopic findings consisted of degeneration/necrosis of hepatocytes, intracytoplasmic eosinophilic inclusions in hepatocytes (Fig. 3A, A2), deposition of pigment in sinusoids, basophilia of hepatocytes, hypertrophy/hyperplasia of bile ducts, and mixed cell infiltrate in portal areas of the liver. Reticulin stain demonstrated a disruption of lobular architecture (Supplementary Fig. SF1B). A prominent dose-dependent increased accumulation of neutral triglycerides and lipids was evidenced by Oil Red O stain (Fig. 3C–F).

Hepatocytes showed minimal to moderate intracytoplasmic inclusions that were positive for glycoprotein using the Periodic Acid Shift (PAS) (Supplementary Fig. SF1A) and PAS diastase histochemical stains, for albumin (Fig. 3B) and complement membrane attack complex (C5b9) IHC (data not shown). No evidence of fibrin accumulation/thrombus formation was observed by phosphotungstic acid-hematoxylin stain and TEM (data not shown).

**Figure 4.**
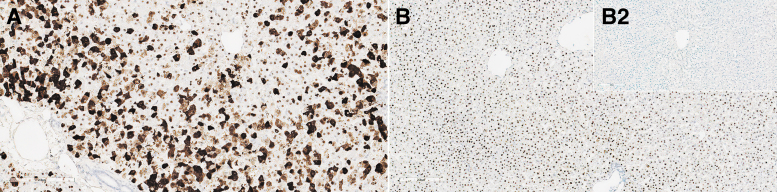
Liver from monkeys: composite figure depicting ISH of *SMN1* in the liver from animals receiving the test article versus control. *SMN1* ISH staining in the liver from female 16, 4 days after a single IV bolus injection of AAV-PHP.B-CBh-*SMN1* at 1 × 10^14^ vg/kg **(A)**: a patchy pattern of intracytoplasmic and intranuclear staining of minimal to marked severity can be observed. *SMN1* ISH in the liver from female 12, 5 days after a single IV bolus injection of AAV-PHP.B-CBh-*SMN1* at 2 × 10^13^ vg/kg **(B)** shows that the staining is essentially confined to hepatocyte nuclei. The corresponding control (female 10) shows no staining **(***insert* **B2)**. ISH, *in situ* hybridization.

In the cynomolgus monkey liver, ISH showed increased labeling/detection of SMN transgene staining in the cytoplasm at ≥5 × 10^13^ vg/kg within hepatocytes (Fig. 4). The effects of RNase and DNase on staining indicated that the cytoplasmic staining was *SMN1* transgene mRNA, and nuclear staining was primarily vector DNA (Supplementary Fig. SF2). Interestingly, despite robust mRNA expression in hepatocyte cytoplasm at ≥5 × 10^13^ vg/kg, IHC-positive staining for *SMN1* transgene protein was found in only a few hepatocytes in these dose groups.

**Figure 5.**
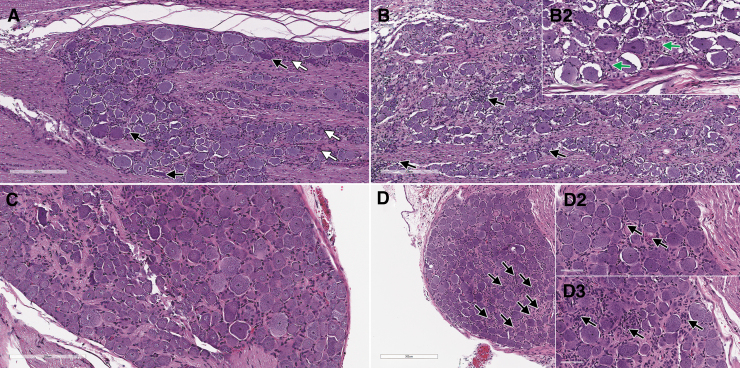
DRG form monkey **(A, B)** and rat **(C, D)**. Dorsal root ganglion of a control cynomolgus monkey (male 1) stained with hematoxylin and eosin, showing the presence of shrunken dark neurons **(A**, *black arrows***)** and mononuclear inflammatory cells **(A**, *white arrows***)**. The dark neurons represent a common technical artifact, while the presence of mononuclear inflammatory cells in DRGs at a low severity is a normal histological feature in the cynomolgus monkey. In the DRG from male 8 treated at 2 × 10^13^ vg/kg, 5 days after a single IV bolus injection of AAV-PHP.B-CBh-*SMN1*, larger focal aggregates of mononuclear cells **(B**, *black arrows***)** and evidences of macrophages surrounding and engulfing neuronal cell bodies (interpreted as neuronophagia) **(***insert* **B2**, *green arrows***)** can be observed. When compared to the normal appearance of a control rat DRG on day 29 in male 25 **(C)**, a low magnification view of a DRG from male 41, 29 days after a single IV bolus injection of AAV-PHP.B-CBh-*SMN1* at 5 × 10^13^ vg/kg, shows that multiple areas seem devoid of normal neuronal cell bodies (*arrows*) **(D)**. At a higher magnification, neuronal degeneration (*arrows*) is characterized by a large and eosinophilic cell body, peripheral displacement or loss of the nucleus, disappearance of Nissl substance that has become condensed in peripheral densely eosinophilic granules (neuronal chromatolysis) **(***insert* **D2)**. Multiple areas are devoid of neurons, but show proliferations of satellite cells (*arrows*) interpreted as Nageotte nodules **(***insert* **D3)**. Neuronal degeneration shows variability across animals and ganglia and is performed in comparison to control slides taken as a reference. DRG, dorsal root ganglia.

**Figure 6.**
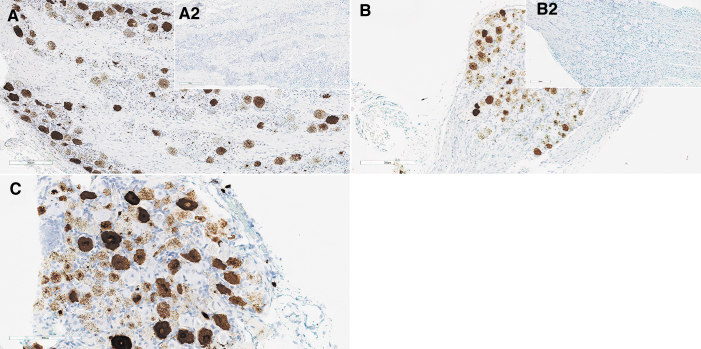
DRG from cynomolgus monkey **(A)** and rat **(B, C)** showing ISH of *SMN1*. *SMN1* ISH of a control cynomolgus monkey (male 2) shows no staining **(***insert* **A2)**, while *SMN1* ISH of male 7, 5 days after a single IV bolus injection of AAV-PHP.B-CBh-*SMN1* at 2 × 10^13^ vg/kg **(A)**, shows a patchy cytoplasmic and nuclear granular staining. Some neurons seem not to be transduced, while others display various degrees of staining intensity, likely reflecting the variety of neuron types within the DRGs and/or transduction variability. *SMN1* ISH in rat DRGs, demonstrating an absence of staining in control male 2 on day 4 **(***insert* **B2)**, a patchy pattern of intracytoplasmic and intranuclear staining of minimal to marked severity in male 21 after a single IV bolus injection of AAV-PHP.B-CBh-*SMN1* at 1 × 10^14^ vg/kg on day 4 **(B)**, persisting on day 29 in male 44 at the same dose level **(C)**.

In the DRGs, minimal focal to multifocal mononuclear cell infiltrate and reactive satellite glial cells were associated with occasional single minimal necrotic neurons (shrunken neurons/neuronophagy) in males at 1 × 10^14^ vg/kg and a single female at 5 × 10^13^ vg/kg (Fig. 5A, B). Positive ISH confirmed the transduction at 1 × 10^14^ vg/kg in some DRG neurons with a patchy pattern (Fig. 6A).

### Wistar Han rat study

Remarkable clinical pathology findings were limited to transient higher group mean monocyte and large unstained cell values (2.7 × and 3.6 × , respectively) at 1 × 10^14^ vg/kg on day 4.

**Figure 7.**
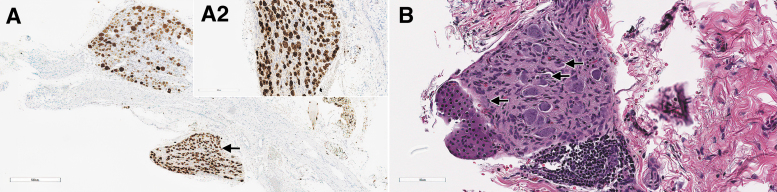
Sympathetic (autonomic) ganglia from rats administered with AAV-PHP.B-CBh-*SMN1* demonstrating *SMN1* ISH staining and morphologic changes. In male 47, on day 29 after a single IV bolus injection of AAV-PHP.B-CBh-*SMN1* at 1 × 10^14^ vg/kg, the section of a DRG shows an adjacent sympathetic ganglion **(A**, *arrow***)** that displays a uniform and strong *SMN1* ISH staining pattern. Higher magnification of this sympathetic ganglion **(***insert* **A2)** shows that all neuronal cell bodies stain positively for *SMN1* by ISH. With hematoxylin and eosin staining, neuronal degeneration/necrosis can be observed in the sympathetic neuron of male 48 on day 29 after a single IV bolus injection of AAV-PHP.B-CBh-*SMN1* at 1 × 10^14^ vg/kg **(B,** *arrows***)**.

On day 29, dose-dependent minimal to mild degeneration/necrosis of neurons in DRGs occurred in treated rats at ≥5 × 10^13^ vg/kg, in association with occasional reactive satellite glial cells and/or minimal to mild infiltration of mononuclear cells (Fig. 5C, D). The cellular infiltrates in DRGs were positively characterized on day 29 by IHC for CD3, and the satellite glial cells for GFAP expression (data not shown). Secondary to neuronal necrosis in rats at ≥5 × 10^13^ vg/kg, there was minimal to moderate degeneration of nerve fibers. Importantly, minimal degeneration/necrosis of neurons in sympathetic paravertebral thoracic ganglia associated with minimal mononuclear cell infiltrate was observed in 3/3 rats at 1 × 10^14^ vg/kg, in which these structures had been incidentally sampled (Fig. 7B).

Dose-dependent minimal to mild degeneration of nerve fibers also occurred in the brain (spinal trigeminal tracts) and spinal cord (dorsal funiculi and spinal nerve roots) in rats at ≥5 × 10^13^ vg/kg.

Nondose-dependent minimal degeneration/necrosis of cardiomyocytes associated with mononuclear cell infiltrate occurred in all rats administered the test article. Minimal to moderate and multifocal degeneration/necrosis of myofibers of the intercostal muscle in the sternum sections occurred in association with minimal to mild mononuclear cell infiltrate in rats at 1 × 10^14^ vg/kg.

Vehicle controls and animals on days 4 and 29 dosed 1 × 10^14^ vg/kg were processed for *SMN1* ISH (Fig. 6B, C), demonstrating intracytoplasmic and intranuclear staining for *SMN1* in sensory neurons at 1 × 10^14^ vg/kg. The staining was finely stippled granular to coalescent and very intense.

*SMN1* ISH performed on sympathetic ganglia also displayed very intense and extensive staining for *SMN1* (Fig. 7A).

**Figure 8.**
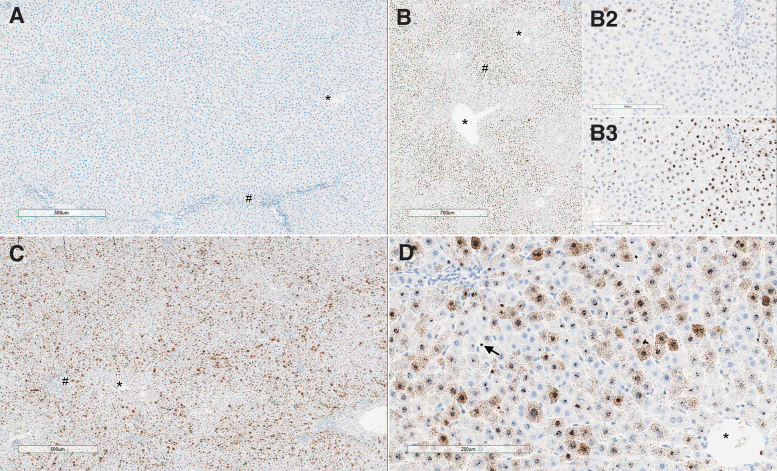
Liver from rats on day 4 and days 28/29 showing comparative staining for ISH of *SMN1* in animal receiving either AAV-PHP.B-CBh-*SMN1* or vehicle. Representative areas corresponding to central veins (*) and periportal areas (#) are identified. Untreated control animal on day 4 **(A)** showing no staining. **(B, B2, B3)** correspond to day 4 rats treated with 1 × 10^14^ vg/kg **(B** at low magnification and *insert* **B3** at higher magnification**)** or 2 × 10^13^ vg/kg **(***insert* **B2)**. Note prominent periportal staining with the *SMN1* ISH probe that is primarily located within hepatocyte nuclei with only rare cytoplasmic staining and less intense staining in hepatocytes around the central vein. **(B2)** Shows a less intense ISH probe staining associated with the lower dose. **(C)** Rat treated with 1 × 10^14^ vg/kg on day 29. Note prominent periportal staining (#) and less intense staining in hepatocytes around the central vein (*). **(D)** Animal treated with 1 × 10^14^ vg/kg on day 28. Higher power view of distribution of staining. Note marked heterogeneity in individual hepatocyte staining. Some hepatocytes only contain nuclear staining and there is variability in the prominence of cytoplasmic staining in hepatocytes, and rare sinusoidal lining cell staining (*arrow*).

In the liver, there was a dose-dependent *SMN1* ISH staining in the cytoplasm and nucleus of periportal to midzonal hepatocytes of animals administered the test article on both days 4 and 29 (Fig. 8), with highest intensity on day 29 at 1 × 10^14^ vg/kg.

**Figure 9.**
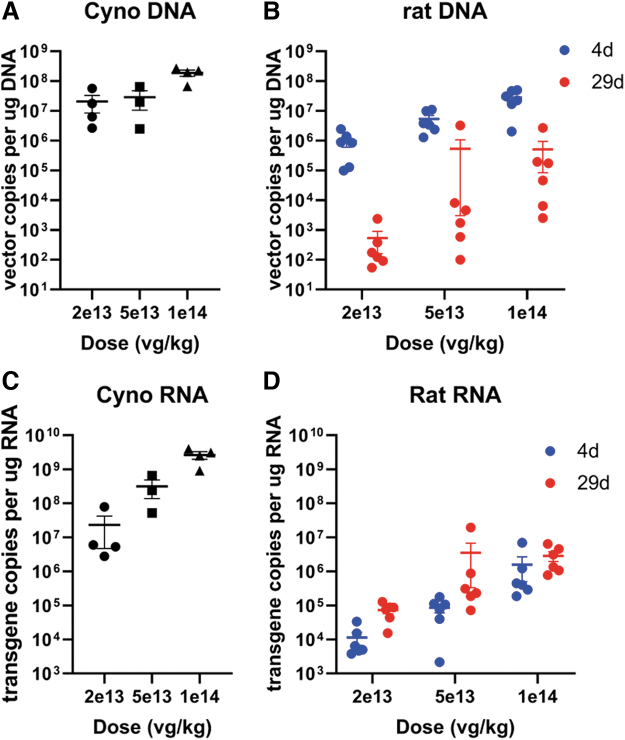
Analysis of vector and transgene copies normalized by total DNA **(A, B)** and RNA **(C, D),** respectively, in the liver from cynomolgus monkeys **(A, C)** and rat **(B, D)** at euthanasia. A comparison across species given the same dose and measured on day 4 (rats) or days 4–5 (cynomolgus monkeys) shows that RNA transgene copies are much higher (∼1,000 × ) in monkeys than in rats, while vector copies appear ∼10 times higher in monkeys than in rats at 2 × 10^13^ vg/kg and within the same range for higher doses.

qPCR performed in the liver on day 4 in the rat showed a clear dose-related uptake of vector. A comparison across species given a similar dose and measured on day 4 (rats) or days 4–5 (cynomolgus monkeys) shows that, although vector copies appear 10 × higher in monkeys than in rats at 2 × 10^13^ vg/kg or within the same range for higher doses, RNA transgene copies were much higher (∼10^3^ × ) in monkeys than in rats, suggesting that transgene copies could be a major driver of toxicity and difference across species (Fig. 9).

RNAseq evaluation of the transgene expression in the liver of the rat and monkey showed a different pattern (Supplementary Fig. SF3). In both species, low coverage near the 3′ end suggests that the expressed transcript is not the intended full-length *SMN1* transgene. In cynomolgus monkeys, the large majority of the reads were in the correct and expected orientation, while rat exhibited a complex mixture of reads in all orientations, mostly unpaired. As cDNA was prepared in the same way, this likely reflects a difference in processing of transcripts in the two species (*e.g.,* potential for forming double-stranded RNA in rat).

## DISCUSSION

The study performed in cynomolgus monkeys administered an AAV9 variant (AAV-PHP.B-CBh-*SMN1*) at 2 × 10^13^, 5 × 10^13^, or 1 × 10^14^ vg/kg per IV route was aligned with the previous report of a single acute toxicity event leading to the euthanasia of 1/1 Rhesus macaque on day 5 following IV administration of an AAV-PHP.B vector at 7.5 × 10^13^ vg/kg.^5^ Early mortality and clinical pathology data triggered the ethical decision to euthanize the remaining animals on days 4–5 and cancel the planned biodistribution evaluation.

An earlier study performed in Rhesus Macaques administered an AAV9 variant (AAVhu68-CBh-*SMN1*) by IV route at 2 × 10^14^ vg/kg had also resulted in acute liver failure in 1/3 animals and transiently elevated transaminases that resolved without clinical sequelae in the remaining 2/3 of the animals.^5^ Altogether, these studies indicate an acute liver toxicity following IV administration of a high dose of AAV9-variant vector in different species of macaques. The early onset of liver failure does not seem compatible with an adaptive cell-mediated or humoral immune response. The marked dose-dependent increase in transgene mRNA with minimal number of hepatocytes that were positive for *SMN1* transgene protein by IHC suggests a direct effect on hepatocyte homeostasis leading to cell death or faulty processing of the transgene mRNA.

The marked dose-dependent increase in neutral lipids is consistent with hepatocyte injury due to several possible hepatocellular malfunctions such as decreased oxidation or use of free fatty acids, impaired synthesis of apoprotein, impaired combination of protein and triglycerides to form lipoproteins, and/or impaired release of lipoproteins from hepatocytes.^12^ The changes in cytokines and complement split products are believed to be secondary to the marked liver injury, because of the close temporal association of increase in these parameters and lack of response in the low-dose group, despite significant liver injury.

The intracytoplasmic eosinophilic inclusions observed in the hepatocytes of monkeys administered test article in this study are morphologically reminiscent of Mallory bodies, although not confirmed by histochemical and immunohistochemical characterization.^13^ These may represent the uptake of plasma proteins that have been described to occur in hypoxic states.^14^ Similar inclusions have also been described as a background finding in other species and are believed to be due to the uptake of plasma proteins, secondary to hemodynamic and hypoxic changes occurring at the time of euthanasia.^15–18^

Decreased platelets and fibrinogen and increased PT and APTT and D-dimers suggested a consumptive coagulopathy, although no sinusoidal fibrin plugs were observed. The animals that were euthanized did not exhibit signs of shock, despite marked increases in liver enzymes and development of coagulopathy, suggesting that the coagulopathy was likely a secondary manifestation of liver tissue injury, with endothelial damage and release of tissue components activating the coagulation cascade.^19^

The markedly greater expression of the *SMN1* transgene mRNA expression in the liver of the cynomolgus monkey compared to the rat and lack of liver toxicity in the rat suggests that the level of transgene expression, even in the absence of protein, may be the primary factor leading to hepatocyte cell death in cynomolgus monkeys.

However, a direct effect of the vector construct or process-related DNA impurities on hepatocytes, independent of transgene expression, and differences in species sensitivity cannot be ruled out. The RNAseq data suggest that the rat and monkey may be processing vector-derived nucleic acids differently and these differences are resulting in both quantitative as well as qualitative differences in transgene mRNA production. The truncation at the 3′ end of the transgene RNA noted in the RNAseq data from both species suggests that there may have been aberrant forms of vector DNA packaged during the production process, which is consistent with recent reports of aberrant DNA sequences that can be packaged during AAV vector production.^20–22^

The use of next-generation sequencing technology to characterize these aberrant forms of co-packaged DNA impurities or chimeric DNA sequences that arise during the production of AAV vectors is an emerging area of AAV vector characterization.^20–22^ The role of these unintended nucleic acid sequences, as well as species differences in their processing, and the induction of cellular injury when high doses of AAV vectors are administered, is an area for further investigation.

An early onset of DRG degeneration/necrosis could be observed in cynomolgus monkeys euthanized on days 4–5. Although not consistently observed across all animals of a given dose, these time-course data, when associated with the simultaneous patchy pattern of ISH detection of the transgene in DRGs, suggest that neuronal degeneration may be an early event resulting from cellular machinery overload by the transgene. These data were aligned with a recent meta-analysis of studies performed with multiple vectors and conducted predominantly in Rhesus macaques, suggesting that acute time points (<14 days) do not show consistent DRG damage across animals.^6^ Of note, *SMN1* IHC performed on cynomolgus monkey DRGs showed there was endogenous expression of *SMN1* in neuronal cell bodies of control DRGs, precluding the assessment of vector-derived *SMN1* expression by this technique (data not shown).

Rats do not reproduce liver toxicity, despite dose-dependent ISH transgene expression in the cytoplasm and nucleus of periportal to midzonal hepatocytes.

A previous report indicated that AAV-PHP.B was neurotropic in C57BL/6J mice.^7^ This study extends observations with the PHP.B vector to rat and demonstrated a pattern of DRG (prominent neuronal degeneration/necrosis accompanied by a low level of inflammation) and secondary sensory tract (brain and spinal cord) toxicity, which shared some commonalities with observations in nonrodent species.^5^ In addition, heart and skeletal muscle necrosis were observed in the rat, suggesting that rats may represent an alternative to large animal species to inform about the nervous and muscular toxicity of AAVs.

ISH distribution of *SMN1* transgene was observed with a mosaic pattern of variable intensity across ganglionic cells of thoracic/lumbar DRGs. A clear relationship between the intensity of transcription and neuronal damage was not evident. In addition to neuronal cell bodies in DRGs, a positive *SMN1* ISH signal was also consistently observed in (incidentally sampled) rat sympathetic paravertebral ganglia neurons that displayed degenerative features similar to those observed in DRGs. The ISH staining appeared very homogeneous across neuronal cell bodies of sympathetic ganglia. To our knowledge, these are the first observations suggesting that the tropism of AAV-PHP.B-CBh-*SMN1* extends to the sympathetic autonomic system and is associated with degenerative changes.

These observations were then confirmed by additional internal data with another AAV and construct (data not published). Considering that the sympathetic autonomic system (like DRGs) is not protected by the blood–brain barrier and may be exposed not only by IV but also by intrathecal administration,^23^ the autonomic system may warrant further attention in future preclinical studies as well as in patient monitoring, and sampling of autonomic system representative ganglia in preclinical studies should be considered.

CD3 IHC in Wistar Han rat DRGs on day 29 demonstrated traces to minimal, focal to multifocal, infiltration of T lymphocytes in the stroma around neurons and nerve fibers, suggesting a possible role of these cells in the pathogenesis of DRG toxicity in the rat.

## CONCLUSION

Our data demonstrate the potential for severe liver toxicity when high doses of AAV are administered intravenously in cynomolgus monkey, which is more commonly used than the Rhesus monkey for pharmaceutical safety evaluation. This study also suggests that AAV-induced acute liver toxicity in the monkey is a direct effect on the hepatocyte following transduction. Some additional work is needed to dissect out the mechanisms leading to cell machinery overwhelming with this construct, at the transcription and/or translation levels. This work is the first to demonstrate that the rat, similar to the mouse^24^ and monkey, is sensitive to DRG toxicity and that autonomic nervous system ganglia may also be a site of AAV-associated neuronal toxicity.

## Supplementary Material

Supplemental data

Supplemental data

Supplemental data

Supplemental data

Supplemental data

Supplemental data

Supplemental data

Supplemental data

Supplemental data

Supplemental data

Supplemental data

Supplemental data

Supplemental data
