# Supplementary material for: Biodistribution and Tolerability of AAV-PHP.B-CBh-SMN1 in Wistar Han Rats and Cynomolgus Macaques Reveal Different Toxicologic Profiles
Source: Hum Gene Ther. 2022 Feb 14;33(3-4):175–87. doi: 10.1089/hum.2021.116 (PMC8885435; doi:10.1089/hum.2021.116)
Supplement: Supplemental data [file Supp_TableS1.docx]

**Supplementary Table S1: Cynomolgus monkeys included in the study**

| **MALES** | | | | | |
| --- | --- | --- | --- | --- | --- |
| **Animal study identification** | **Body weight on Day 1 (kg)** | **Date of birth** | **Age on Day 1** | **Neutralizing Antibody Status** | **Binding Antibody** |
| 001 | 4.6 | 06/Jun/16 | 3 years, 3 months | - | - |
| 002 | 3.5 | 12/Apr/17 | 2 years, 4 months | - | - |
| 003 | 3.1 | 11/Nov/16 | 2 years, 10 months | - | - |
| 004 | 4.2 | 27/Feb/16 | 3 years, 6 months | - | - |
| 005 | 4 | 09/Feb/16 | 3 years, 7 months | - | - |
| 006 | 3.2 | 17/Apr/17 | 2 years, 4 months | - | + |
| 007 | 4.1 | 22/Feb/16 | 3 years, 6 months | - | - |
| 008 | 3.1 | 15/Jun/16 | 3 years, 3 months | - | + |

| **FEMALES** | | | | | |
| --- | --- | --- | --- | --- | --- |
| **Animal study identification** | **Body weight on Day 1 (kg)** | **Date of birth** | **Age on Day 1** | **Neutralizing Antibody Status** | **Binding Antibody** |
| 009 | 4.4 | 01/Sep/15 | 4 years | - | - |
| 010 | 3 | 14/Feb/16 | 3 years, 6 months | - | - |
| 011 | 3.6 | 24/Sep/15 | 3 years, 11 months | - | - |
| 012 | 3.4 | 24/Sep/15 | 3 years, 11 months | - | - |
| 013 | 4.3 | 02/Sep/15 | 4 years | - | - |
| 014 | 4.9 | 03/Feb/16 | 3 years, 7 months | nAb=1:5 | + |
| 015 | 3.3 | 23/Sep/15 | 3 years, 11 months | - | - |
| 016 | 3.2 | 15/Feb/16 | 3 years, 6 months | nAb=1:5 | + |

All animals selected for the study were seronegative (titer < 1:5) for neutralizing antibodies to the AAV-PHP.b capsid in order to ensure unaltered tissue transduction. AAV-PHP.b capsid binding antibodies were also evaluated and animals that were considered negative or positive were distributed within treatment groups to spread any potential effect on transduction.

All animals (including controls) were instrumented with telemetry transmitters (Data Sciences International [DSI] telemetry implant with femoral artery catheter and biopotential leads) and had a period of at least 2 weeks of post-operative recovery prior to the experimental start date.
