# Supplementary material for: Biodistribution and Tolerability of AAV-PHP.B-CBh-SMN1 in Wistar Han Rats and Cynomolgus Macaques Reveal Different Toxicologic Profiles
Source: Hum Gene Ther. 2022 Feb 14;33(3-4):175–87. doi: 10.1089/hum.2021.116 (PMC8885435; doi:10.1089/hum.2021.116)
Supplement: Supplemental data [file Supp_TableS2.docx]

**Supplementary Table S2: Experimental design for Cynomolgus study**

| **Experimental Design** | | | | | | |
| --- | --- | --- | --- | --- | --- | --- |
| **Group Number** | **Test Article**  **Number or Vehicle** | **Dose (vg/kg)^a^** | **Concentration**  **(vg/mL)** | **Dose Volume**  **(mL/kg) ^b^** | **Animal Numbers** | |
|  |  |  |  |  | **Males** | **Females** |
| 1 | Vehicle | 0 | 0 | 2 | 1-2 | 9-10 |
| 2 | AAV-PHP.b-CBh-SMN1 | 2x10^13^ | 1x10^13^ | 2 | 3-4 | 11-12 |
| 3 | AAV-PHP.b-CBh-SMN1 | 5x10^13^ | 2.5x10^13^ | 2 | 5-6 | 13-14 |
| 4 | AAV-PHP.b-CBh-SMN1 | 1x10^14^ | 0.5x10^14^ | 2 | 7-8 | 15-16 |
| a. All doses are expressed as vg of vector genomes per kg of body weight.  b. The dose volume is based on the most recent scheduled body weight. | | | | | | |
