# Supplementary material for: Biodistribution and Tolerability of AAV-PHP.B-CBh-SMN1 in Wistar Han Rats and Cynomolgus Macaques Reveal Different Toxicologic Profiles
Source: Hum Gene Ther. 2022 Feb 14;33(3-4):175–87. doi: 10.1089/hum.2021.116 (PMC8885435; doi:10.1089/hum.2021.116)
Supplement: Supplemental data [file Supp_TableS3.docx]

**Supplementary Table S3: Experimental design for rat study**

| **Experimental Design** | | | | | | | | |
| --- | --- | --- | --- | --- | --- | --- | --- | --- |
| **Group Number** | **Test Article**  **Number or Vehicle** | **Dose (vg/kg)^a^** | **Concentration**  **(vg/mL)** | **Dose Volume**  **(mL/kg) ^b^** | **Male Numbers** | | | |
|  |  |  |  |  | **Day 4** | | **Day 29** | |
|  |  |  |  |  | **1^st^ Cohort** | **2^nd^ Cohort** | **1^st^ Cohort** | **2^nd^ Cohort** |
| 1 | Vehicle | 0 | 0 | 5 | 1-3 | 4-6 | 25-27 | 28-30 |
| 2 | AAV-PHP.b-CBh-SMN1 | 2x10^13^ | 0.4x10^13^ | 5 | 7-9 | 10-12 | 31-33 | 34-36 |
| 3 | AAV-PHP.b-CBh-SMN1 | 5x10^13^ | 1x10^13^ | 5 | 13-15 | 16-18 | 37-39 | 40-42 |
| 4 | AAV-PHP.b-CBh-SMN1 | 1x10^14^ | 2x10^13^ | 5 | 19-21 | 22-24 | 43-45 | 46-48 |
| a. All doses are expressed as vg of vector genomes per kg of body weight.  b. The dose volume is based on the most recent scheduled body weight. | | | | | | | | |
