# Supplementary material for: Biodistribution and Tolerability of AAV-PHP.B-CBh-SMN1 in Wistar Han Rats and Cynomolgus Macaques Reveal Different Toxicologic Profiles
Source: Hum Gene Ther. 2022 Feb 14;33(3-4):175–87. doi: 10.1089/hum.2021.116 (PMC8885435; doi:10.1089/hum.2021.116)
Supplement: Supplemental data [file Supp_TableS4.docx]

**Supplementary Table S4: Hematology, coagulation, clinical chemistry, cytokines and other biomarkers evaluated in monkeys**

| **Hematology and coagulation parameters** | |
| --- | --- |
| Red Blood Cells (RBC) | Red Cell Distribution Width (RDW) |
| Hemoglobin (HGB) | Reticulocytes (RETIC) |
| Hematocrit (HCT) | Platelets (PLT) |
| Mean Cell Volume (MCV) | Mean Platelet Volume (MPV) |
| Mean Cell Hemoglobin (MCH) | White Blood Cells (WBC) |
| Mean Cell Hemoglobin Concentration (MCHC) | White Cell Differential |
| Activated Partial Thromboplastin Time (APTT) | Prothrombin Time (PT) |
| Fibrinogen (FIB) |  |
|  | |

| **Clinical chemistry parameters** | |
| --- | --- |
| Alanine Aminotransferase (ALT) | Albumin (ALB) |
| Globulin (GLOB) | Globulin (GLOB) |
| Albumin/Globulin Ratio (AG) | Albumin/Globulin Ratio (AG) |
| Blood Urea Nitrogen (BUN) | Blood Urea Nitrogen (BUN) |
| Creatinine (CREA) | Creatinine (CREA) |
| Phosphorus (PHOS) | Phosphorus (PHOS) |
| Calcium (CA) | Calcium (CA) |
| Sodium (NA) | Sodium (NA) |
| Potassium (K) | Potassium (K) |
| Albumin (ALB) | Chloride (CL) |
| C Reactive Protein (CRP) |  |

| **Serum cytokines** | |
| --- | --- |
| Interleukin 6 (IL6) | Interferon-γ-induced protein 10 (IP10) |
| Interleukin 10 (IL10) | Monocyte Chemoattractant Protein 1 (MCP1) |
| Interferon alpha 2a (IFNα-2a) | Tumor Necrosis Factor Alpha (TNFa) |
| Interferon beta (IFNβ) |  |

| **Other biomarkers** | |
| --- | --- |
| Complement C5b-9 | Complement C4a |
| Complement C3a | Complement Bb |
