# Supplementary material for: Biodistribution and Tolerability of AAV-PHP.B-CBh-SMN1 in Wistar Han Rats and Cynomolgus Macaques Reveal Different Toxicologic Profiles
Source: Hum Gene Ther. 2022 Feb 14;33(3-4):175–87. doi: 10.1089/hum.2021.116 (PMC8885435; doi:10.1089/hum.2021.116)
Supplement: Supplemental data [file Supp_TableS5.docx]

**Supplementary Table S5: Hematology, coagulation, clinical chemistry, cytokines and other biomarkers evaluated in rats**

| **Hematology and coagulation parameters** | |
| --- | --- |
| Red Blood Cells (RBC) | Red Cell Distribution Width (RDW) |
| Hemoglobin (HGB) | Reticulocytes (RETIC) |
| Hematocrit (HCT) | Platelets (PLT) |
| Mean Cell Volume (MCV) | Mean Platelet Volume (MPV) |
| Mean Cell Hemoglobin (MCH) | White Blood Cells (WBC) |
| Mean Cell Hemoglobin Concentration (MCHC) | White Cell Differential |
| Activated Partial Thromboplastin Time (APTT) | Prothrombin Time (PT) |
| Fibrinogen (FIB) |  |
|  | |

| **Clinical chemistry parameters** | |
| --- | --- |
| Alanine Aminotransferase (ALT) | Albumin (ALB) |
| Aspartate Aminotransferase (AST) | Globulin (GLOB) |
| Alkaline Phosphatase (ALP) | Albumin/Globulin Ratio (AG) |
| Gamma‑Glutamyltransferase (GGT) | Blood Urea Nitrogen (BUN) |
| Glutamate Dehydrogenase (GLDH) | Creatinine (CREA) |
| Bilirubin, Total (TBIL) | Phosphorus (PHOS) |
| Cholesterol (CHOL) | Calcium (CA) |
| Triglyceride (TRIG) | Sodium (NA) |
| Glucose (GLUC) | Potassium (K) |
| Total Protein (TP) | Chloride (CL) |
| Serum indices for hemolysis, icterus, and lipemia will be performed. | |

| **Serum cytokines** | |
| --- | --- |
| Interleukin 6 (IL6) | Monocyte Chemoattractant Protein 1 (MCP1) |
| Interferon-γ-induced protein 10 (IP10) | Tumor Necrosis factor Alpha (TNFa) |

| **Other biomarkers** | |
| --- | --- |
| Complement C5b-9 |  |
