# Supplementary material for: Biodistribution and Tolerability of AAV-PHP.B-CBh-SMN1 in Wistar Han Rats and Cynomolgus Macaques Reveal Different Toxicologic Profiles
Source: Hum Gene Ther. 2022 Feb 14;33(3-4):175–87. doi: 10.1089/hum.2021.116 (PMC8885435; doi:10.1089/hum.2021.116)
Supplement: Supplemental data [file Supp_TableS7.docx]

**Supplementary Table S7: Tissues evaluated in rats**

| **Tissues Collected** | **Organs Weighed**  **(All Dose Groups)** | **Tissues Processed for Slide Preparation (X)**  **Scheduled Necropsy**  **Dose Group** | | | |
| --- | --- | --- | --- | --- | --- |
|  |  | **Group 1** | **Group 2** | **Group 3** | **Group 4** |
| Bone Marrow, Right Femur^a,b^ |  |  |  |  |  |
| Bone Marrow, Sternum |  | X |  |  | X |
| Brain | X | X |  |  | X |
| Dorsal Root Ganglion^b,c^ |  | X |  |  | X |
| Epididymis |  | X |  |  | X |
| Eye |  | X |  |  | X |
| Heart |  | X |  |  | X |
| Kidney |  | X |  |  | X |
| Liver^b^ | X | X | X | X | X |
| Lung |  | X |  |  | X |
| Lymph Node, Mesenteric^b^ |  | X |  |  | X |
| Macroscopic Findings |  | X | X | X | X |
| Mesentery |  | X |  |  | X |
| Pancreas |  | X |  |  | X |
| Small Intestine, Duodenum |  | X |  |  | X |
| Small Intestine, Jejunum |  | X |  |  | X |
| Spinal Cord |  | X |  |  | X |
| Spleen^b^ |  | X |  |  | X |
| Stomach |  | X |  |  | X |
| Testis |  | X |  |  | X |
| Thymus |  | X |  |  | X |
| a. Push-through collection method.  b. Processed to paraffin in 72 hours to enable ISH.  c. Collect one pair from each level: cervical, thoracic, and lumbar. | | | | | |
