# Supplementary material for: Biodistribution and Tolerability of AAV-PHP.B-CBh-SMN1 in Wistar Han Rats and Cynomolgus Macaques Reveal Different Toxicologic Profiles
Source: Hum Gene Ther. 2022 Feb 14;33(3-4):175–87. doi: 10.1089/hum.2021.116 (PMC8885435; doi:10.1089/hum.2021.116)
Supplement: Supplemental data [file Supp_TableS9.docx]

**Supplementary Table S9: immunohistochemistry procedure**

| **Antibody** | **Clone** | **Vendor** | **Catalog Number** | **Isotype** | **Stock Concentration** | **Dilution** | **Pretreatment** |
| --- | --- | --- | --- | --- | --- | --- | --- |
| C5b-9 | Polyclonal | Abcam | Ab55811 | Rabbit IgG | 1mg/ml | 1/100 | Protease 8min at 37°C |
| Cytokeratin 8 | Polyclonal | Abcam | Ab175249 | Rabbit IgG | 1.08mg/ml | 1/1500 | Epitope Retrieval 2 20 minutes |
| Pan Cytokeratin | Polyclonal | Abcam | Ab9377 | Rabbit IgG | 1.21mg/ml | 1/200 | Epitope Retrieval 2 20 minutes |
| Albumin | Polyclonal | Abcam | Ab2406 | Rabbit IgG | 1mg/ml | 1/2000 | Epitope Retrieval 1 20 minutes |
| Hsp90 | C45G5 | Cell Signaling | 4877 | Rabbit IgG | 20ug/ml | 1/400 | Epitope Retrieval 1 20 minutes |
| SMN1 | 2B1 | Abcam | Ab55831 | Mouse IgG1 | 1mg/ml | 1/200 | Epitope Retrieval 1 20 minutes |
| Ubiquitin | Polyclonal | Cell Signaling | 3933S | Rabbit IgG | 71ug/ml | 1/500 | Epitope Retrieval 1 20 minutes |
| CD3 | Polyclonal | Abcam | Ab5690 | Rabbit IgG | 200ug/ml | 1/1000 | Epitope Retrieval 2 20 minutes |
| GFAP | E4L7M | Cell Signaling | 80788 | Rabbit IgG | 30.5ug/ml | 1/250 | Epitope Retrieval 1 20 minutes |
