# Supplementary material for: Biodistribution and Tolerability of AAV-PHP.B-CBh-SMN1 in Wistar Han Rats and Cynomolgus Macaques Reveal Different Toxicologic Profiles
Source: Hum Gene Ther. 2022 Feb 14;33(3-4):175–87. doi: 10.1089/hum.2021.116 (PMC8885435; doi:10.1089/hum.2021.116)
Supplement: Supplemental data [file Supp_TableS10.docx]

**Supplementary table S10: Treatment-related clinical pathology findings in ALT, AST, platelets, fibrinogen, PT, APTT, D-Dimers and albumin in Cynomolgus monkeys.**

|  | Dose (vg/kg) | | | | | | | | | | | | | | | |
| --- | --- | --- | --- | --- | --- | --- | --- | --- | --- | --- | --- | --- | --- | --- | --- | --- |
|  | 0 | | | | 2E13 | | | | 5E13 | | | | 1E14 | | | |
| Study Day | Alanine Aminotransferase (reference range: 23-118 U/L): | | | | | | | | | | | | | | | |
| Predose | 34 | 30 | 54 | 44 | 36 | 37 | 24 | 45 | 41 | 72 | 46 | 26 | 32 | 58 | 71 | 49 |
| Day 1 | 76 | 34 | 63 | 41 | 43 | 41 | 38 | 48 | 74 | 79 | 59 | 27 | 61 | 51 | 72 | 64 |
| Day 3 | 72 | 33 | 56 | 35 | 1697 | 312 | 135 | 242 | 776 | 291 | 674 | 299 | 346 | 496 | 193 | 1253 |
| Day 4 | 70 | 43 | 57 | 34 | 2008 | 700 | 467 | 1116 | 1119 | N/A | 2629 | 1173 | 2124 | 738 | 1992 | 2608 |
|  | Aspartate Aminotransferase (reference range: 25-64 U/L): | | | | | | | | | | | | | | | |
| Predose | 37 | 35 | 26 | 44 | 41 | 31 | 37 | 33 | 39 | 43 | 40 | 26 | 40 | 29 | 44 | 30 |
| Day 1 | 103 | 48 | 54 | 54 | 44 | 41 | 50 | 36 | 79 | 92 | 49 | 35 | 66 | 32 | 57 | 39 |
| Day 3 | 54 | 35 | 33 | 44 | 1725 | 226 | 168 | 240 | 671 | 258 | 522 | 251 | 231 | 503 | 162 | 944 |
| Day 4 | 54 | 36 | 35 | 41 | 376 | 251 | 214 | 618 | 408 | N/A | 1002 | 360 | 907 | 566 | 1633 | 1050 |
|  | Total Protein (reference range: 6.9-8.9 g/dL): | | | | | | | | | | | | | | | |
| Predose | 7.0 | 7.8 | 7.6 | 7.5 | 6.9 | 6.8 | 7.4 | 7.8 | 7.3 | 8.0 | 8.0 | 7.7 | 7.6 | 7.3 | 7.3 | 7.4 |
| Day 1 | 7.0 | 7.8 | 7.0 | 7.2 | 6.9 | 6.8 | 7.0 | 7.7 | 7.3 | 7.5 | 8.0 | 7.4 | 7.5 | 7.4 | 7.1 | 7.4 |
| Day 3 | 7.3 | 7.7 | 7.1 | 7.1 | 7.0 | 6.9 | 7.1 | 7.8 | 7.4 | 6.7 | 7.9 | 7.2 | 7.4 | 7.2 | 6.8 | 7.6 |
| Day 4 | 6.8 | 7.6 | 7.2 | 6.9 | 7.0 | 6.9 | 7.0 | 7.8 | 7.2 | N/A | 7.9 | 7.0 | 6.2 | 6.2 | 5.3 | 6.9 |
|  | Albumin (reference range: 3.8-5.0 g/dL): | | | | | | | | | | | | | | | |
| Predose | 4.7 | 4.8 | 4.7 | 4.5 | 4.6 | 4.4 | 4.6 | 4.4 | 4.5 | 4.9 | 4.6 | 4.6 | 4.9 | 4.8 | 4.1 | 4.6 |
| Day 1 | 4.7 | 4.9 | 4.4 | 4.4 | 4.7 | 4.4 | 4.3 | 4.4 | 4.6 | 4.8 | 4.7 | 4.5 | 4.9 | 4.9 | 4.1 | 4.7 |
| Day 3 | 4.9 | 4.8 | 4.5 | 4.3 | 4.6 | 4.4 | 4.4 | 4.4 | 4.6 | 4.2 | 4.6 | 4.4 | 4.7 | 4.7 | 3.9 | 4.7 |
| Day 4 | 4.5 | 4.8 | 4.6 | 4.3 | 4.6 | 4.3 | 4.2 | 4.4 | 4.3 | N/A | 4.5 | 4.2 | 3.9 | 4.1 | 3.0 | 4.2 |
|  | Prothrombin time (reference range: 11.4-13.6 sec): | | | | | | | | | | | | | | | |
| Predose | 12.3 | 12.2 | 13.0 | 12.1 | 12.7 | 12.1 | 13.2 | 12.8 | 13.1 | 12.7 | 12.7 | 12.6 | 12.1 | 13.1 | 12.6 | 12.5 |
| Day 1 | 13.3 | 12.1 | 13.3 | 11.9 | 12.4 | 12.2 | 13.4 | 13.0 | 13.6 | 13.6 | 12.9 | 13.4 | 13.0 | 13.0 | 13.7 | 12.1 |
| Day 3 | 13.0 | 12.2 | 12.6 | 12.1 | 17.8 | 14.4 | 15.3 | 14.7 | 24.4 | 25.7 | 21.0 | 17.6 | 23.5 | 20.9 | 24.7 | 24.0 |
| Day 4 | 12.0 | 11.9 | 12.5 | 11.9 | 15.6 | 13.3 | 16.1 | 15.3 | 35.5 | N/A | 16.7 | 15.3 | 27.6 | 48.8 | 89.2 | 24.2 |
|  | Activated Partial Thromboplastin time ( reference range: 19.6-25.8 sec): | | | | | | | | | | | | | | | |
| Predose | 22.2 | 22.2 | 20.8 | 23.1 | 22.0 | 22.6 | 22.7 | 21.9 | 22.4 | 21.5 | 20.9 | 21.2 | 21.7 | 24.4 | 23.3 | 21.3 |
| Day 1 | 23.1 | 22.1 | 20.3 | 22.8 | 21.6 | 22.1 | 22.0 | 21.8 | 22.4 | 23.3 | 20.6 | 21.5 | 22.3 | 23.4 | 23.6 | 20.7 |
| Day 3 | 21.9 | 21.8 | 19.5 | 23.0 | 25.8 | 25.7 | 23.3 | 24.5 | 30.5 | 37.5 | 28.6 | 26.6 | 33.1 | 35.4 | 35.5 | 33.3 |
| Day 4 | 21.3 | 21.8 | 19.1 | 21.9 | 24.4 | 24.5 | 23.9 | 25.5 | 35.5 | N/A | 26.6 | 26.9 | 34.5 | 49.9 | 66.1 | 33.8 |
|  | Fibrinogen (reference range: 141-281 mg/dL): | | | | | | | | | | | | | | | |
| Predose | 288 | 267 | 238 | 224 | 203 | 239 | 203 | 196 | 183 | 219 | 220 | 195 | 233 | 201 | 202 | 163 |
| Day 1 | 292 | 268 | 254 | 223 | 240 | 247 | 219 | 203 | 168 | 143 | 204 | 212 | 245 | 224 | 191 | 184 |
| Day 3 | 326 | 293 | 312 | 212 | 233 | 251 | 252 | 219 | 176 | 133 | 202 | 220 | 236 | 211 | 160 | 174 |
| Day 4 | 304 | 280 | 308 | 202 | 204 | 256 | 226 | 226 | 121 | N/A | 191 | 243 | 163 | 90 | 60 | 119 |
|  | D-dimer (reference range: 0.27-2.36 ug/mL): | | | | | | | | | | | | | | | |
| Predose | 2.49 | 1.43 | 0.85 | 1.78 | 0.87 | 2.00 | 0.87 | 0.56 | 0.91 | 1.73 | 0.77 | 0.72 | 0.81 | 0.93 | 1.21 | 0.68 |
| Day 1 | 1.88 | 1.18 | 1.21 | 1.01 | 1.05 | 1.98 | 0.87 | 0.60 | 0.56 | 2.79 | 0.72 | 0.80 | 0.88 | 0.48 | 0.72 | 0.70 |
| Day 3 | 2.41 | 1.15 | 1.02 | 1.00 | 2.44 | 2.12 | 0.92 | 0.62 | 1.25 | 2.23 | 1.21 | 0.88 | 1.03 | 1.03 | 1.44 | 1.25 |
| Day 4 | 1.88 | 1.24 | 2.04 | 1.05 | 2.76 | 2.54 | 1.43 | 1.13 | 2.59 | N/A | 2.39 | 1.54 | 2.66 | 2.66 | 3.20 | 2.24 |
|  | Platelets (reference range: 234-598 x10e3/uL): | | | | | | | | | | | | | | | |
| Predose | 579 | 651 | 393 | 402 | 324 | 383 | 537 | 438 | 183 | 437 | 332 | 471 | 437 | 372 | 321 | 626 |
| Day 1 | 458 | 524 | 363 | 334 | 352 | 367 | 556 | 395 | 350 | 339 | 299 | 459 | 360 | 362 | 338 | 521 |
| Day 3 | 498 | 567 | 370 | 353 | 301 | 397 | 556 | 413 | 276 | 208 | 259 | 366 | 306 | 151 | 295 | 403 |
| Day 4 | 509 | 549 | 387 | 341 | 308 | 377 | 541 | 384 | 212 | N/A | 295 | 274 | 60 | 24 | 98 | 134 |

Individual data are given at baseline (predose) and on Days 1, 3 and 4. Reference ranges validated at the test facility are indicated for each parameter. Treatment with a single IV bolus injection of AAV-PHP.B-CBh-SMN1 led to decreased platelets, fibrinogen and albumin, and increased ALT, AST, PT, APTT, D-dimers, ALT, AST.
