# Supplementary material for: Biodistribution and Tolerability of AAV-PHP.B-CBh-SMN1 in Wistar Han Rats and Cynomolgus Macaques Reveal Different Toxicologic Profiles
Source: Hum Gene Ther. 2022 Feb 14;33(3-4):175–87. doi: 10.1089/hum.2021.116 (PMC8885435; doi:10.1089/hum.2021.116)

Supplementary Figure SF1: composite figure depicting the morphologic changes observed microscopically in the liver from monkeys receiving the test article versus controls. (A) Liver from Female 15, 4 days after a single IV bolus injection of AAV-PHP.B-CBh-SMN1 at 1x10^14^ vg/kg, showing intracytoplasmic Periodic Acid Schiff (PAS) positive granules (arrow). (B) Liver from Female 16 stained with Reticulin, showing collapse of reticulin scaffold (arrows) and confirming extensive necrosis (insert B2 corresponds to the liver of a control animal similarly stained with reticulin).


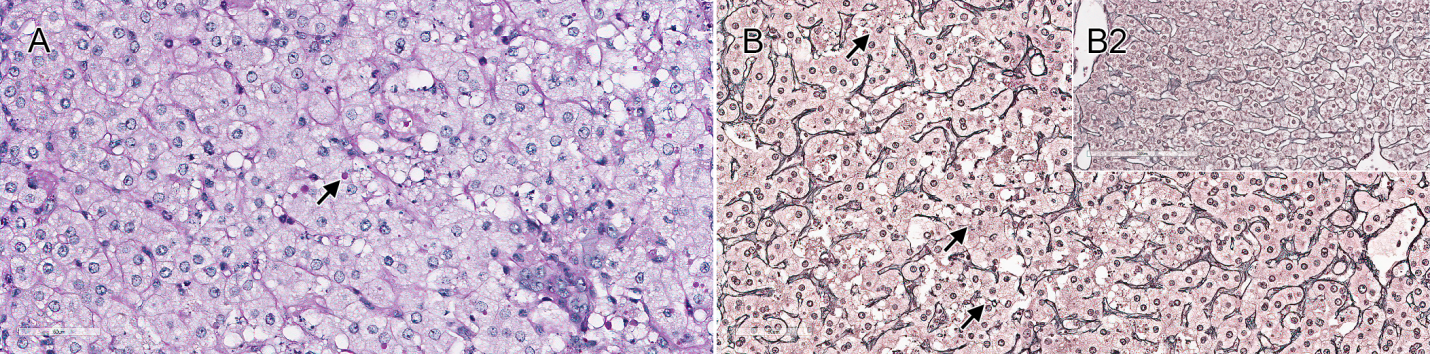


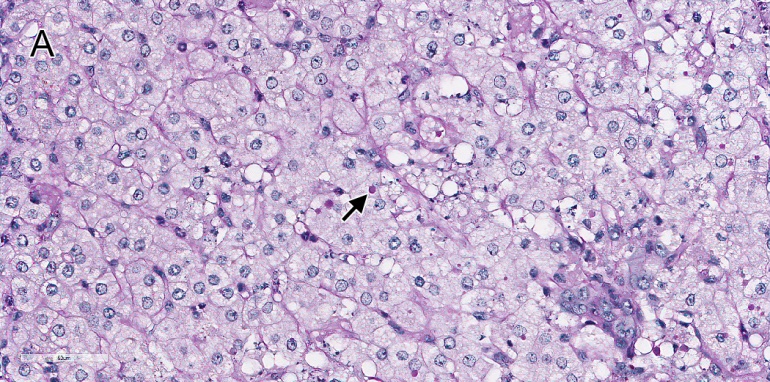


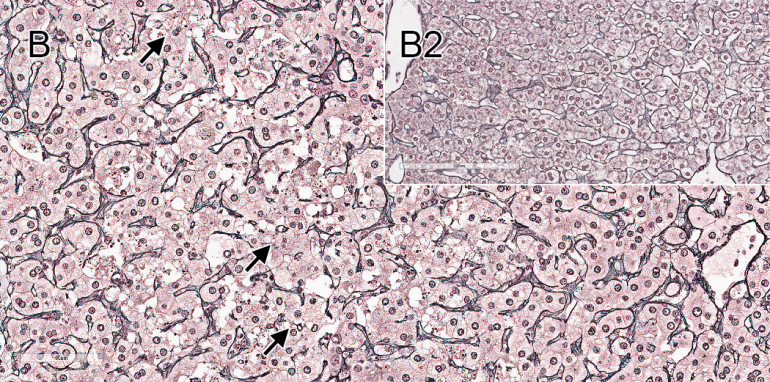

Supplement: Supplemental data [file Supp_FigS1.docx]
