# Supplementary material for: Biodistribution and Tolerability of AAV-PHP.B-CBh-SMN1 in Wistar Han Rats and Cynomolgus Macaques Reveal Different Toxicologic Profiles
Source: Hum Gene Ther. 2022 Feb 14;33(3-4):175–87. doi: 10.1089/hum.2021.116 (PMC8885435; doi:10.1089/hum.2021.116)

Supplementary Figure SF2: composite figure demonstrating the subcellular location of vector DNA (nucleus) and RNA (cytoplasmic) by ISH in the liver from Female 13 administered a single IV bolus injection of AAV-PHP.B-CBh-SMN1 at 5x10^13^vg/kg, following DNase or RNase pretreatment. Low and high magnification (A and B respectively) of liver without pretreatment. DNase pretreatment results in the loss of nuclear staining while retaining cytoplasmic staining (C). RNase pretreatment results in the loss of cytoplasmic staining while retaining nuclear staining (D).


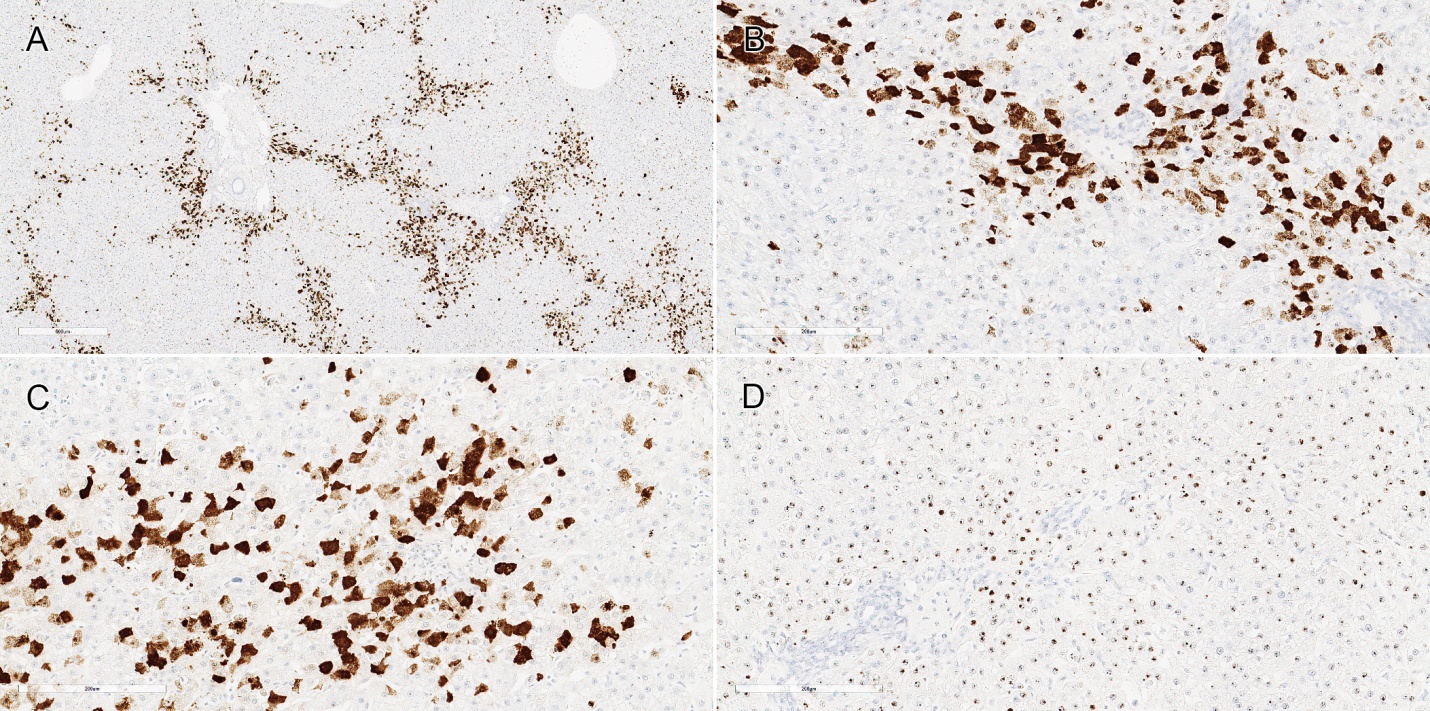


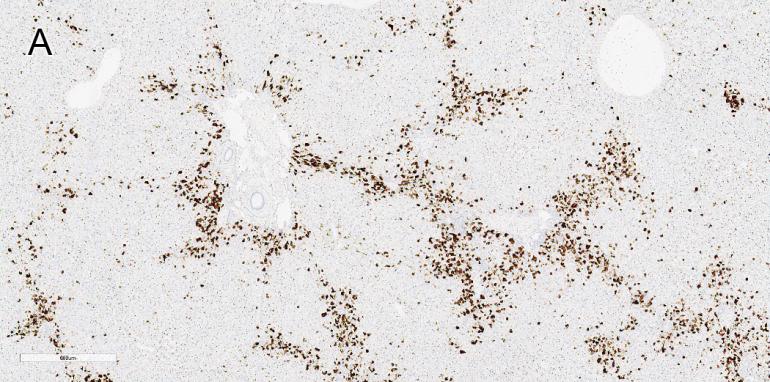


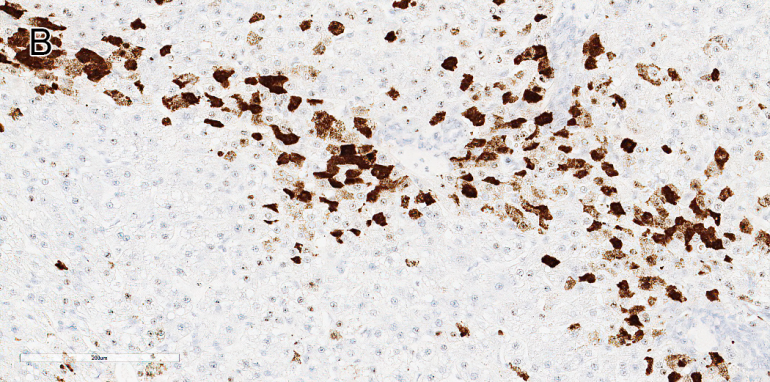


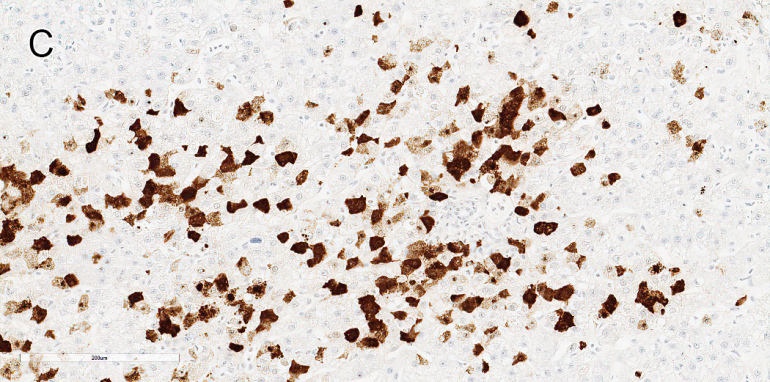


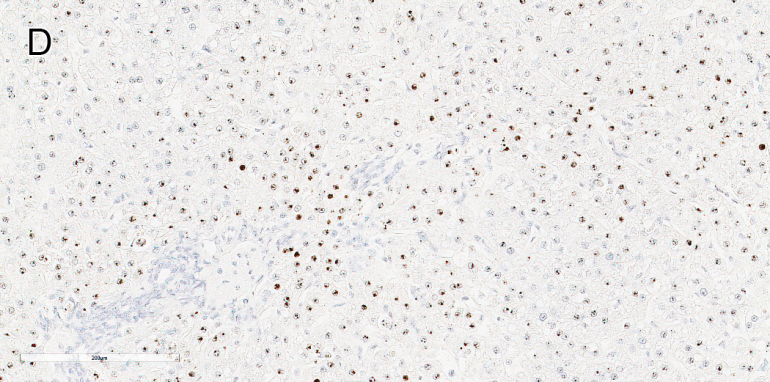

Supplement: Supplemental data [file Supp_FigS2.docx]
