# Supplementary material for: Biodistribution and Tolerability of AAV-PHP.B-CBh-SMN1 in Wistar Han Rats and Cynomolgus Macaques Reveal Different Toxicologic Profiles
Source: Hum Gene Ther. 2022 Feb 14;33(3-4):175–87. doi: 10.1089/hum.2021.116 (PMC8885435; doi:10.1089/hum.2021.116)

Supplementary Figure SF3: Stranded RNASeq coverage and individual sequence read alignment to the SMN1 transgene for liver samples from high dose rat on Day 4 (upper panel) and cynomolgus monkey (lower panel). The histogram represents the sum of reads at a particular location (5’ to 3’ base pair location listed on the upper x-axis). Cyan represents reverse paired reads (the expected orientation for TruSeq stranded libraries), red represents reverse unpaired reads, and green represents forward unpaired reads. Both species show low coverage in a region near the 3’ end of the transgene, but only the rat sequence shows a high proportion of reads that are not mapping as expected, either showing up as forward reads or unpaired reads.


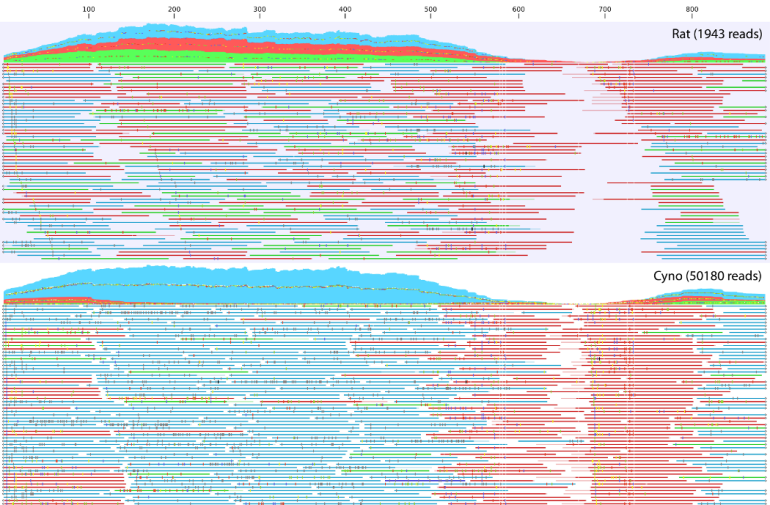

Supplement: Supplemental data [file Supp_FigS3.docx]
